# Screening with a limited safety net: post-screening service gaps and inequitable access to child protection in Vietnam

**DOI:** 10.3389/fpubh.2026.1930066

**Published:** 2026-10-21

**Authors:** Thi Minh Le, Phuong Thi Bui, Tho Nguyen

**Affiliations:** 1 Faculty of Health Social Sciences, Hanoi University of Public Health, Hanoi, Vietnam; 2 International Research Institute for Artificial Intelligence and Digital Transformation, Dong A University, Da Nang, Vietnam

**Keywords:** access to services, child protection, health equity, help-seeking, institutional trust, mental health services, post-screening service gaps, Vietnam

## Abstract

**Background:**

Violence against children is a population-level public health problem in Vietnam, and schools are increasingly tasked with detecting it; yet detection protects a child only if it leads to services. In an earlier phase of this research, about one-third of Vietnamese adolescents who had experienced physical abuse had told someone, yet only 2.95% had told a formal recipient (a teacher, school counsellor, school social worker, or health worker). The problem therefore lies at the formal output of the system, not in children’s silence. This study maps post-screening service gaps in the national child-protection system and analyzes students’ trust as the mechanism linking those gaps to disclosure and help-seeking, read against the 2025 administrative reform.

**Methods:**

In a convergent design, we combined a national desk review of the child-protection and mental-health service system with a qualitative study comprising 25 in-depth interviews and 16 focus group discussions with teachers and students in eight lower-secondary schools in Bac Giang province (December 2023–March 2024). The qualitative strand illustrates mechanisms rather than representing the national population.

**Results:**

The desk review shows that the chain of screening, referral, and support breaks or weakens at each major link, that the only near-universal formal channel is the national child helpline, and that coordination is dispersed across ministries operating different management models. In the interviews, students trusted individuals — mainly peers and friends — more than institutions, and disclosures that went unanswered left institutional trust unreinforced. Because no non-stigmatizing mental-health service existed locally, a referral led only to the stigmatized provincial psychiatric hospital, so students avoided the “mental” label rather than take up help.

**Conclusion:**

The 2025 reform weakened coordination in the short term but could, if implemented well, embed social-work services within the health system. These findings suggest that screening should be expanded only alongside the services to which children can be referred. To our knowledge, this is the first study to map the post-screening gap in Vietnam and tie it to institutional trust. The findings are relevant to Sustainable Development Goal 3 (good health and well-being) and Goal 10 (reduced inequalities).

## 1 Background

Violence against children is a population-level public health problem in Vietnam rather than a marginal or localized one. The 2014 Multiple Indicator Cluster Survey found that 68.4% of children aged 1–14 had experienced violent discipline and 58.2% had experienced psychological aggression (1, 2). Such exposure is consistently associated with adverse consequences for children’s physical health, mental health, and development (3, 4), which makes the systems responsible for detecting and responding to it a matter of public health, not only of child welfare.

Policy attention in schools has concentrated on the first step of that response — identifying affected children — and screening and early-detection activities are being scaled up across the school system. Detection, however, does not by itself protect a child, and the core problem is not that children stay silent. In an earlier phase of this research, around one-third of Vietnamese adolescents who had experienced physical abuse had disclosed it to someone, yet only 2.95% had disclosed it to a formal recipient, such as a teacher, school counsellor, school social worker, or healthcare provider (5). Low formal disclosure matters because the formal tier is where a child could be connected to care — and that need is substantial. National data show the scale of unmet mental-health need: in the 2013 Global School-based Student Health Survey, 14.0% of students aged 13–15 had seriously considered suicide in the previous year (6). Even when a need is recognized, only 8.4% of adolescents with mental-health problems reach services (7). In the baseline survey for the present project, 25.2% of students screened positive for depressive symptoms on the CESD-R (unpublished data), underscoring the scale of need in this setting. These national surveys, conducted in 2013 and 2014, remain the most recent nationally representative estimates of their kind for Vietnam; in the absence of newer national data, and with no evidence that the underlying burden has fallen (8), they continue to describe the baseline against which the current service system must be judged. Within a continuum-of-care logic, screening is only the opening link in a longer chain; what happens after screening — whether a child is referred, seen, followed up and told what happened next — determines whether identification is converted into protection and care. Framed this way, the problem is fundamentally one of access to services, and therefore of equity: whether children who are identified can actually obtain the protection and mental-health support to which they are entitled.

Research on child maltreatment in Vietnam and comparable settings has concentrated on other parts of the problem — the prevalence of abuse, its risk factors, the predictors of disclosure, and school-based counselling (9, 10) — rather than on the post-screening stage. Comparatively little work has examined the post-screening stage itself, or children’s own trust in the institutions meant to help them once they have spoken, and this stage is rarely framed as a service-access equity problem. Yet trust is central: whether disclosure translates into help-seeking depends on the responses a child anticipates, and whether an institution retains a child’s trust depends on whether it reliably responds (11).

This concern is not unique to Vietnam. Child maltreatment is common across Southeast and East Asia, where achievement-oriented family norms and limited child mental-health infrastructure are widespread (12, 13), and many low- and middle-income systems in the region are scaling up school-based detection of mental-health and protection needs faster than they build the referral and follow-up capacity that detection presupposes (14). The post-screening gap examined here is therefore likely to be a regional, not only a national, problem.

The Vietnamese context makes these gaps consequential. The Law on Children and its implementing Decree 56/2017 distribute responsibility for child protection across agencies (15, 16), a national child helpline (111) provides the most visible formal channel, and early detection in schools is actively promoted. On paper, the framework is comprehensive; in practice, implementation is uneven, and the gap is sharpest in schools. Although a school-counselling or “school psychology” function is formally required, in the context of public-sector staff reductions, it is usually carried out by teachers with a few days of training, or by administrative staff, rather than by qualified clinical psychologists or social workers. Children therefore seldom meet a trained professional they can turn to — a structural reason why institutional trust is thin. Provision at the child level, however, is thin and fragmented: mental-health and psychosocial services are governed across several ministries, each with its own management model, and child-specific capacity is limited, so the path from detection to treatment is narrow (17). A broad administrative reform in 2025 then merged provincial units, abolished the district administrative tier, and moved child-related functions towards the health sector (18). This reshaped the coordination architecture and risked removing a coordinating layer without integrating the services beneath it, though it may also open a longer-term opportunity to embed social-work support within the health system.

Against this background, this study maps post-screening service gaps at the level of the national child-protection system and analyzes students’ trust as the mechanism linking those structural gaps to disclosure and help-seeking. We use the 2025 reform in the study province (Bac Giang; in the July 2025 administrative reform, the province was merged into and renamed Bac Ninh) as a forward-looking implication for that gap, since the fieldwork predates it. Two questions guide the study: (a) where does the national post-screening service chain break, and how is it fragmented across governance lines? and (b) how do students and teachers experience this gap, and how does it affect trust? By locating the structural cause in the absence of a trustworthy, available and non-stigmatizing service at the output — rather than in children’s reluctance to speak — the study connects child protection to public health and to the equity agenda of Sustainable Development Goal 3 (good health and well-being) and Goal 10 (reduced inequalities). To our knowledge, this is the first study to map the post-screening service gap in Vietnam’s child-protection system and to link it explicitly to institutional trust as the mechanism, read against the 2025 administrative reform. This study draws on the qualitative, second phase of a research programme on the role of schools in child protection. The first, survey phase produced a companion study on the low formal disclosure of child physical abuse (5); a further study on the lifetime prevalence of child maltreatment, and a study on household and academic determinants of cumulative maltreatment are currently under review. Other outputs address distinct questions on the same primary data: a multi-level stigma and equity analysis of disclosure and a teacher–social-worker dual-role analysis. This study poses a distinct question, with its own analysis about the post-screening stage, service access, and trust at the system level.

## 2 Conceptual framework

We frame the post-screening problem through two primary lenses — the help-seeking pathway and institutional trust — which together locate the problem at the service output and serve as the deductive scaffold for our analysis. The framework is assembled from established theory rather than devised *de novo*: the help-seeking lens draws on staged models of help-seeking (19, 20) and on the ecological, transactional account of disclosure (21, 22), while the trust lens draws on the concept of epistemic trust (11) and, at the service tier, on the stigma construct of label avoidance (23, 24). We combine these into a single scaffold running from a child’s recognition of a problem to the institutional response that either reinforces or erodes trust. Two further ideas, disclosure and stigma-driven label avoidance, are treated not as separate theories but as components of these two lenses.

The help-seeking pathway. Help-seeking is a sequence: recognizing a problem, disclosing it, forming an intention to seek help, and accessing a source (19, 20). Detection is only its first step, and the chain breaks at the final step when the only remaining channel is remote or unfamiliar. Disclosure is the pivotal, selective link in this pathway: it is not a single act but an ecological process shaped by the responses a child anticipates from those around them (21, 22), and children rarely disclose to formal services. When the person causing harm is a parent or caregiver, the salient barriers sit at the family level — loyalty, fear of consequences, the sense that this is a private matter, and normalization of harm — so a system that detects without a reliable route onward stalls the pathway at its most fragile point.

Institutional trust. Whether a child returns to a source of help depends on epistemic trust — trust in the reliability of the support an institution provides (11). Interpersonal trust in a specific adult is distinct from trust in the institution; when disclosure produces no visible response, the feedback loop that would build institutional trust is broken, and such trust is left unreinforced rather than actively destroyed. Trust is further shaped at the service tier by stigma. Label avoidance describes the tendency to avoid a service in order to avoid the label attached to it (23, 24); in this study, it deters uptake of a mental-health referral even where a service exists.

Read together, the two lenses frame our question: the help-seeking pathway locates where the system fails to convert detection into care, and institutional trust explains why those failures accumulate into a durable withdrawal from formal help. The service gap is the structural cause; trust is the mediating mechanism.

## 3 Materials and methods

### 3.1 Study design

This was a convergent, health-systems study with a qualitative core. The qualitative component was descriptive and interpretive in orientation: it stayed close to participants’ own accounts of how detection, referral and support work in practice, rather than testing a hypothesis or building formal theory (25). This orientation suits our aim — to understand how a documented service gap is experienced and how it bears on trust — and it pairs naturally with the desk review in a convergent design, in which two complementary strands are analyzed separately and then brought together to answer one question (26). It combined a national desk review of secondary documentary sources on the child-protection and mental-health service system with primary qualitative data from the second (qualitative) phase of a larger study of the role of schools in child protection, conducted in eight schools and comprising in-depth interviews with school actors and focus group discussions with students. The two strands were chosen to answer complementary questions and were integrated by design: the desk review identifies where the post-screening service chain is structurally broken, while the qualitative interviews explain how those structural gaps are experienced by teachers and students and how they bear on trust. The desk review is therefore the main analytic object and the level at which system-wide claims are made; the qualitative strand illustrates mechanisms and is not intended to be representative of students in Vietnam. Throughout, the design prioritizes transferability over statistical generalization.

### 3.2 Data sources

*National desk review*. We conducted a structured document review — a desk-based, narrative synthesis of publicly available policy, administrative and statistical sources rather than a systematic review of primary studies — to map the post-screening service chain at the system level. In a desk review of this kind, the analyst assembles and interprets existing authoritative documents (laws, decrees, official statistics and agency reports) to reconstruct how a system is designed to work and where, in practice, it breaks down, without generating new primary data. Sources were purposively identified from four types of repositories. The first two were Vietnamese legislation and ministry documents (the National Assembly legal database and the portals of the Ministries of Health, of Labour – Invalids and Social Affairs, and of Education and Training) and national survey reports (the Multiple Indicator Cluster Survey, the Global School-based Student Health Survey, and the Viet Nam National Adolescent Mental Health Survey). The remaining two were international-agency reports on Vietnam (UNICEF, WHO, UNFPA, UN Women and the Overseas Development Institute) and operational statistics of the national child helpline. We included documents that were national in scope, publicly available, and directly relevant to detection, referral, mental-health or social-work provision, or coordination for children; we excluded purely clinical guidance, sub-national pilots, and non-authoritative web content. Searches of these repositories used terms relating to child protection, mental-health and social-work provision, and to screening, referral, follow-up and inter-agency coordination, and titles and document summaries were screened against the inclusion criteria; the core authoritative sources retained, with their governance line and relevance, are listed in Supplementary Table S1. From each source we extracted, by governance line, what the system provides on paper, where it breaks in practice, and the implication for the help-seeking pathway. The review was carried out in 2024 alongside the fieldwork; extractions were charted, by governance line, into the service-gap map (Table 1) and were read against the interview accounts, so that documentary claims and lived experience could be triangulated. Documents relating to the 2025 reform (18) were reviewed separately to inform the forward-looking implications discussed in Section 5, rather than as part of the service-gap mapping.

**Table 1:** A map of post-screening service gaps by governance line (national-system level).

Link in the chain	On paper (national)	Where it breaks in practice	Implication for trust
Screening (schools)	Schools are tasked with detection; many have a psychological counseling room	No professional social worker; no standard bridge to services outside the school	The entry point fails to create a trustworthy pathway out
Tier 1: health – mental health	A nationwide examination and treatment system for mental health	Thin child-specific facilities and workforce; a narrow path from detection to treatment; the “mental illness” label	Avoided even where it exists (label avoidance)
Tier 2: psychosocial – social work – social protection	Child affairs agency, commune-level officials, national helpline (111)	Commune staff under-professionalized; the 111 helpline rarely refers onward to specialized services; no professional clearly follows the case	Unclear who follows the case (“I told someone but do not know where to go”)
Cross-tier coordination	Inter-agency coordination mechanism	Dispersed across ministries with different management models; coordination exists mainly on paper	No coherent institution in which to place trust

Compiled by the authors from the national desk review (Section 3.2); the full list of sources is given in Supplementary Table S1.

*Primary qualitative interviews and focus groups*. As part of a research project funded by NAFOSTED (Vietnam) and carried out between December 2023 and March 2024, we generated primary qualitative data in eight lower-secondary schools — four rural and four urban — in Bac Giang province, a northern province with mixed lowland, midland and mountainous terrain; the province was subsequently merged into the enlarged Bac Ninh in 2025. The dataset comprised 25 in-depth interviews (8 school-board members, 8 homeroom teachers, 8 teachers who doubled as counsellors or social-work focal points, and 1 leader of the provincial Department of Education and Training) and 16 focus group discussions with students (eight groups in grade 7 and eight in grade 9) (Table 2), recruited by purposive sampling for their direct or observed experience relevant to screening, referral and support in schools. Participants were recruited through written invitation letters and took part voluntarily after written informed consent (Section 3.5); only invited individuals participated. The interviews and focus groups were conducted by two female researchers — one holding a PhD and the other a Master’s degree, both experienced in qualitative research — using a semi-structured guide informed by the two conceptual lenses; sessions lasted approximately 45–60 min — a length consistent with the focused, semi-structured guide and, for the student focus groups, with the participants’ age — were not repeated, involved no third parties, and were accompanied by field notes. Notably, none of the schools employed a dedicated school social worker or professional counsellor; these a question distinct from the project’s other outputs that draw on the same primary dataset: where a companion study analyses the cultural and stigma dynamics that keep adolescents silent, and the phase-1 study quantifies disclosure rates, this study analyzes the post-screening service system and the institutional-trust consequences of its gaps. The interviews overlap, but the analytic question, framing and conclusions do not. As the sample was purposive and bounded to specific settings, the findings illustrate mechanisms rather than estimate their prevalence. We judged the material adequate for this purpose on the basis of information power (27) — the interviews were specific to the phenomenon, theoretically informed and analyzed against an established framework — while remaining explicit about the limits of what such data can claim. Because the fieldwork predates the 2025 reform, the interviews describe the pre-reform system; the reform itself is analyzed through the desk review.

**Table 2:** Characteristics of interview (IDI) participants and student focus-group discussions (FGD).

Participant group	Method (units)	Sex/composition
School-board members	IDI (8 interviews)	All male
Homeroom teachers	IDI (8 interviews)	7 female, 1 male
Teacher-counsellors / social-work focal points	IDI (8 interviews)	All female
Provincial education-authority leader (Department of Education and Training)	IDI (1 interview)	1 (sex not disclosed)
Students, grade 7	FGD (8 groups)	Mixed-sex
Students, grade 9	FGD (8 groups)	Mixed-sex

*Research team and reflexivity*. Data were collected in person by two female Vietnamese researchers holding Master’s and doctoral degrees, trained in qualitative methods and familiar with the local cultural context. They were not affiliated with the participating schools, an arrangement intended to reduce the influence of the school environment on what participants felt able to say. We kept a reflexive record of the researchers’ positions — as public-health and social-work researchers external to the schools — and considered how these might shape the questions asked and the interpretations made; this reflexivity is carried into the analysis (Section 3.4).

*Data collection*. Semi-structured guides were adapted to each participant group: teachers were asked about their responsibilities, knowledge, and experience in detecting and responding to maltreatment, while students reflected on their understanding of harm, trust in adults, and perceived barriers to disclosure. Sessions were held in private rooms on school premises, with no third party present, and lasted approximately 45–60 min. All sessions were audio-recorded and transcribed verbatim, and transcripts were reviewed by the researchers for accuracy. Transcription and coding were conducted in Vietnamese; quotations selected for reporting were translated into English using a back-translation procedure to ensure semantic equivalence. No repeat interviews were carried out. Transcripts were not returned to participants. However, at the end of each session the researcher summarized the main points and invited the participant to comment on, correct or add to this summary, providing an opportunity for immediate respondent validation.

### 3.3 Analysis

The desk review was analyzed as a service-gap mapping exercise, organized by governance line, to identify where and how the chain is fragmented. The interview data were analyzed using reflexive thematic analysis following the six-phase, reflexive approach of Braun and Clarke (28, 29), in an abductive mode: deductive codes were derived from the two conceptual lenses (the help-seeking pathway and institutional trust) and complemented by inductive codes developed from the data. Coding was undertaken by two researchers who had also conducted the interviews; consistent with reflexive thematic analysis, coding and theme development were treated as an interpretative, collaborative process rather than a reliability exercise, with interpretive differences resolved through discussion. Coding was done manually, with Mindjet MindManager used to organize codes and build the thematic structure; the resulting coding framework, with overarching themes, subthemes, and representative codes, is provided as Supplementary Table S2. Theme development was iterative and continued until the team judged that the material carried sufficient information power (27). The two strands were then read against each other so that the qualitative themes could explain the trust implications of the gaps identified in the desk review.

The two analyses were then integrated in a convergent, side-by-side design. The service-gap map derived from the desk review provided the structural frame, and the qualitative themes were read against it, so that each documented break in the post-screening chain could be matched to how it was experienced by school actors and students and to its consequences for trust. Points of convergence and divergence between the two strands are drawn together in the Results (Table 1; Figure 1) and revisited in the Discussion.

**Figure 1:**
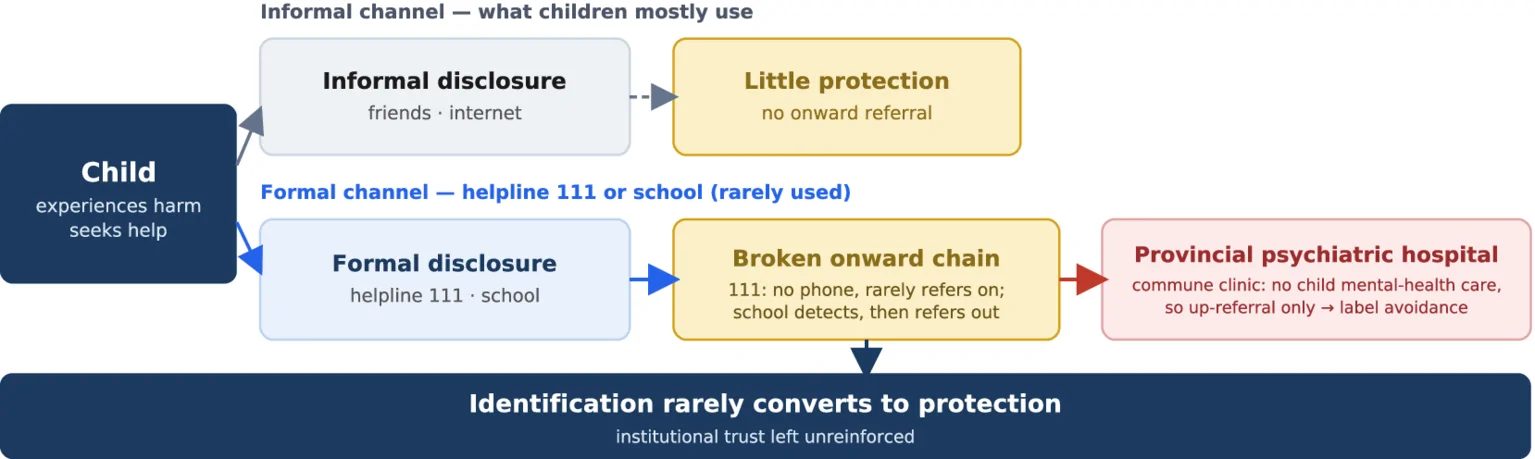
The post-screening help-seeking pathway in this setting. Children reach help through two channels. The informal channel (friends or the internet), used most often, leads nowhere formal and yields little protection. The formal channel (helpline 111 or the school) is rarely used and its onward chain is broken: referral runs only to the single, distant provincial psychiatric hospital, which is stigmatized and avoided (label avoidance, shown in red). Identification therefore rarely converts to protection, leaving institutional trust unreinforced.

### 3.4 Trustworthiness and reporting

To strengthen rigor, we followed the criteria of Lincoln and Guba (30): triangulation across the desk review and interview strands; reflexive dialogue between coders; an audit trail documenting analytic decisions; respondent validation through the end-of-interview summaries (Section 3.2); and a reflexivity statement on the researchers’ positions. Consistent with the study’s aim, we sought transferability rather than statistical generalization, and we report the qualitative component in line with the COREQ checklist (31), provided as Supplementary material.

### 3.5 Ethics

The study obtained ethical approval and informed consent, such as child assent and parental consent (Research Ethics Committee of Hanoi University of Public Health, Vietnam; approval No. 321/YTCC-HD3). All quotations are anonymized, and any potentially identifying details are removed or presented in composite form. Data on children were handled under the study’s data-protection arrangements. A distress and safeguarding protocol was in place. Interviewers were trained to pause or end a session if a participant became distressed and to signpost support. Where a disclosure indicated that a child might be at risk of harm, a pre-agreed referral procedure was followed through the school’s designated child-protection focal point and, where appropriate, the national child helpline.

## 4 Results

### 4.1 A national map of post-screening service gaps

Read as a system, the intended pathway is a chain: a school screens or detects a concern, refers the child onward, the child is seen and supported, and the outcome is fed back so that the case is followed to resolution. The desk review indicates that this chain is broken or weakly resourced at each major link and that the breaks compound one another (Figure 1; Table 1). In Table 1, the national desk review supplies the “on paper” picture, while the “in practice” column combines it with the qualitative accounts. As these come from a single province, they illustrate how each link fails rather than establishing how often it does.

At the entry point, schools are tasked with detection, and many operate a psychological counselling room, but they typically lack a professional social worker and any standardized bridge to services outside the school, so detection frequently has nowhere reliable to lead. Along the health line, a nationwide mental-health treatment system exists on paper, but child-specific facilities and workforce are thin, and the path from detection to treatment is narrow (17); the “mental illness” label further deters use even where a service is available. The psychosocial and social-protection line runs through the child affairs agency, commune-level officials, and the national helpline. The helpline receives several hundred thousand calls a year (368,444 in 2022 and 323,615 in 2023) (32) but rarely refers callers onward to specialized or provincial services (33). According to the 111 helpline’s own reporting, only a small share of contacts — around 2–5% — are escalated to emergency referral to local child-protection services, such as commune authorities, the police, health facilities or social-work centers (32). Support along this line is likewise thin, commune-level staff are under-professionalized, and no single professional is clearly responsible for following a case once it leaves the school. Across all lines, coordination is dispersed among ministries operating different management models, so inter-agency coordination exists largely on paper. The net effect is that no single actor is accountable for what happens after screening.

### 4.2 How the gaps are experienced: qualitative themes

The interviews illustrate how these structural gaps are experienced and why they bear on trust. The themes are presented to explain mechanisms, and not to estimate their frequency; all quotations are anonymized. We organized the material under the study’s two conceptual lenses as two overarching themes — the help-seeking pathway, where disclosure stalls at its pivotal link, and institutional trust, including stigma and label avoidance — each comprising several subthemes, labeled A–E below and summarized in Table 3.

**Table 3:** Overarching themes, subthemes and illustrative quotations from the interviews and focus groups.

Subtheme	Illustrative quotation
Overarching theme 1 — Help-seeking pathway: disclosure stalls at its pivotal link	
Subtheme C1 — Family-level barriers (parent is the source of harm)	“We do not usually talk about it unless it gets really bad… It’s hard to talk about family stuff.” (FGD, students)
Subtheme E — Disclosure rebounds; the formal response stalls	“We knew the child was being beaten, but the parents came to the school and caused a scene, insisting that it was their own family’s business.” (IDI, teacher)
Overarching theme 2 — Institutional trust (with stigma and label avoidance)	
Subtheme A — No safe channel to disclose or refer on	“…our school does not have a counselling or medical room… no student has ever reported to us about their situation at home.” (IDI, teacher)
Subtheme B — “Speaking up changes little” (unanswered disclosure)	“…even if we did speak up, there’s usually no one who can really help.” (FGD, students)
Subtheme D — Trusting people, not the system	“Students in rural areas may have limited access to… child-protection hotlines such as 111… the lack of other support services makes it difficult for them to disclose.” (IDI, teacher-counsellor)
Subtheme C2 — Label avoidance (the “mental” label)	“Friends would laugh if they knew I went there.” (FGD, students)

#### 4.2.1 Overarching theme 1 — help-seeking pathway: disclosure stalls at its pivotal link

This theme brings together the accounts in which disclosure — the pivotal link in the help-seeking pathway — is suppressed or turned back before it can become a formal case. The shared pattern is that the pathway breaks at or before disclosure: family-level loyalties and the sense that harm is a private matter keep children from speaking when the person causing harm is a caregiver (Subtheme C1), and, where a disclosure is made, it can rebound on the child while the formal response stalls (Subtheme E).

Subtheme C1 — Barriers to disclosure when the person causing harm is a parent or caregiver. The salient barriers here were located at the family level — loyalty, fear of consequences, the sense that this is a private family matter, and normalization of the harm — rather than stigma. Students explained: “We don’t usually talk about it unless it gets really bad, and even then only with close friends. It’s hard to talk about family stuff.” (FGD, students) Normalization was captured by a teacher: “Vietnamese has a saying: spare the rod, spoil the child […] which leads many students to normalise parental hitting or equate physical punishment with proper child-rearing.” (IDI, teacher).

Subtheme E — When disclosure rebounds and the formal response stalls. Disclosure could also make the discloser’s position worse. One teacher recounted that after a student reported violence at home, the family blamed the school, leaving the teacher in a difficult position: under the law, parents remain the child’s legal guardian, which limits what a school can do without the family’s cooperation. As one teacher put it, “We knew the child was being beaten, but the parents came to the school and caused a scene, insisting that it was their own family’s business.” (IDI, teacher) Participants also noted that abuse was often acknowledged only at the severe end: “child abuse is typically recognized only when it involves more severe or extreme actions […] only in cases of serious physical incidents, or when it occurs in families with stepparents or complicated family dynamics.” (IDI, school director) When physical or emotional abuse reached support units or the police, cases frequently stalled because there was no visible bodily evidence to substantiate them, so they were not pursued (33). The formal pathway could therefore not only fail to help but expose the child, and the adults who tried to help, to further difficulty.

#### 4.2.2 Overarching theme 2 — institutional trust (with stigma and label avoidance)

This theme brings together the accounts in which the institution fails to act as a trustworthy, available destination, so trust is withdrawn from it and reinvested in particular people. The shared pattern is that absent, unanswered, or stigmatizing responses leave institutional trust unreinforced: there is no safe channel to disclose or refer on (Subtheme A), speaking up is felt to change little (Subtheme B), trust is placed in specific individuals rather than the system (Subtheme D), and the one mental-health service is avoided because of the label it carries (Subtheme C2).

Subtheme A — No safe channel to disclose or refer on. Participants located the failure in the institution itself: the school offered no trustworthy, standing channel through which a child could disclose or be referred onward. There was often no counselling space and no professional to receive a concern, so neither a disclosure nor any onward step had a reliable destination. As one teacher put it: “We are teachers, and our school does not have a counselling or medical room. Teachers are not formally trained to provide psychological support or counselling; we can only grasp the situation when students choose to share with us […] no student has ever reported to us about their situation at home.” (IDI, teacher) A school director described how the role itself had been hollowed out: “In theory, a school psychology office is required; however, due to the policy of streamlining staff, the role of school counsellor is currently taken on by the Youth Union teacher […] far from professional.” (IDI, school director).

Subtheme B — “Speaking up changes little.” Students recounted instances in which a disclosure had been made, but little had followed — accounts of others’ experience rather than of a case the researchers interviewed directly, yet consistent in portraying institutions as unlikely to respond. This is consistent with institutional trust that is left unreinforced rather than actively destroyed: “It’s hard to tell anyone, because no one wants to say bad things about their own parents. And anyway, even if we did speak up, there’s usually no one who can really help.” (FGD, students).

Subtheme D — Trusting people, not the system. Participants placed trust in specific individuals — typically peers and friends — rather than in institutions or formal channels, and described how a weak position made it hard to reach help independently, even through the national helpline: “Students in rural areas may have limited access to information about self-protection or to child-protection hotlines such as 111. Even when the hotline is introduced, the lack of other support services makes it difficult for them to disclose incidents that occur within the family.” (IDI, teacher-counsellor) A further, concrete barrier was that lower-secondary students typically did not own a phone, so contacting 111 meant using a parent’s device — impractical in general, and unworkable where a parent was the source of the harm.

Subtheme C2 — Avoiding the referral because of the “mental” label. Distinct from C1, a further barrier operated at the service tier: reluctance to take up a mental-health referral because of the label attached to it. In Vietnamese, the clinical term for mental illness carries strong connotations of being “crazy,” so being sent to a mental-health service risks that label; as one student said, “Friends would laugh if they knew I went there.” (FGD, students) This was reinforced by what such a service looked like locally: the study province had a single provincial psychiatric hospital, which participants understood to serve mainly people with severe conditions such as schizophrenia, and they were afraid to be associated with it. For students, simply hearing that they might be taken to the psychiatric hospital was frightening; they associated it with people seen as “crazy,” and it was in any case some distance away. Distance thus compounded the label: the one formal mental-health facility was both stigmatized and hard to reach. Critically, there was no low-stigma alternative within reach: district and provincial general hospitals lacked clinical or pediatric-psychology units, so the single specialized psychiatric hospital was in effect the only formal mental-health destination. With no non-stigmatizing option, seeking help and accepting the “mental” label had become the same choice, and families avoided both. Students also did not see maltreatment as a problem serious enough to warrant a psychiatric hospital, so they turned instead to informal sources or the internet rather than to a formal mental-health service. This is consistent with population-level evidence that stigma leaves people reluctant to approach mental-health services, which are in any case associated mainly with severe disorders (17, 34). Label avoidance therefore deters uptake even where a service exists, and it channels help-seeking away from the formal system.

Read against the service-gap map (Table 1), these themes show how each structural break is experienced on the ground and converts into a loss of trust. The missing bridge out of school (Subtheme A) leaves disclosure unanswered (Subtheme B); the stigmatized, distant mental-health tier is avoided (Subtheme C2); the helpline and commune tier are not trusted to follow a case (Subtheme D); and the formal pathway can rebound on the child and the adults who help (Subtheme E). The structural breaks are thus not experienced as neutral administrative gaps: each unanswered disclosure signals to a child that speaking up may not reliably lead to help, and personalized trust tends to become a substitute for absent institutional trust.

## 5 Discussion

This study set out to locate where Vietnam’s post-screening service chain breaks and to understand how those breaks bear on students’ trust. Two findings stand out. First, at the level of the national system, the chain is broken at every major link, and no single actor is accountable for what happens after a child is identified. Second, in the accounts of students and teachers, this structural gap is not experienced as a neutral administrative shortfall: it signals to children that speaking up may not reliably lead to help, and it tends to push trust away from institutions and onto particular individuals.

Read together, the two strands support a single interpretation. The problem is not primarily that children are unwilling to disclose; indeed, around one-third of adolescents who had experienced physical abuse disclosed to someone, yet almost none reached a formal recipient (5). It is that the environment a child anticipates after disclosure is largely empty, and this anticipated emptiness is itself a barrier to disclosure and help-seeking (21, 22). When an institution does not visibly respond, it does not earn the epistemic trust that would make a child return to it; institutional trust is therefore not reinforced, and personalized trust substitutes for it (11). Where a mental-health referral does exist, the label attached to it can deter uptake — a distinct barrier that operates at the service tier, not at the point of disclosure (23, 24). These mechanisms are analytically separable, but in a fragmented system they compound.

These findings both confirm and extend prior research. That only 2.95% of adolescents who had experienced physical abuse in this project reached a formal recipient (5) is consistent with international evidence that children disclose selectively and seldom to formal services (21, 22); this is especially so for intrafamilial abuse, where loyalty, secrecy and the normalization of physical punishment suppress disclosure — patterns our participants described directly (Subthemes B and C1). Our results add a structural reason for this pattern: even a child who does reach a formal actor finds no reliable route onward (Subtheme A). This resonates with help-seeking research in which structural and informational barriers, not only reluctance, keep young people from care (19, 20), and with Vietnamese system analyses documenting fragmented, unregulated referral and follow-up spread across ministries (17, 33). The label avoidance around mental-health services in our data echoes stigma research on how anticipated labeling deters care-seeking (23, 24) and matches Vietnamese evidence that formal mental-health services are associated with severe illness and carry heavy community stigma (17, 34); in our setting, this was compounded by distance to the single provincial psychiatric facility, so that access was geographic as well as attitudinal (Subtheme C2). Finally, disclosure could rebound on children and on the teachers who tried to help them, through parents’ standing as legal guardians and a reliance on physical evidence. This matches analyses of a child-protection and justice system in which such evidence is rarely available and parental discretion is wide (33) (Subtheme E).

It might be objected that this describes poor access rather than a failure of trust — that children avoid services because these are scarce, distant or unfamiliar, not because they distrust them. Practical and attitudinal barriers are certainly entangled in our data. Yet trust does independent work: disclosure that meets no response erodes trust even where a channel exists (Subtheme B); the mental-health tier is avoided on account of its label even where it is reachable (Subtheme C2); and children route around the formal system toward friends and the internet rather than simply going without help (Subthemes A and D). Access and trust are therefore better read as compounding than as competing explanations, which is why widening access alone — more screening, another hotline — would not, on its own, restore use.

Theoretically, the study extends an established account of disclosure and help-seeking (19–22) by locating the key gap not in the child’s willingness to speak but in the system’s capacity to respond. It also extends epistemic trust (11) beyond the therapeutic dyad to the institutional level: when the post-screening chain repeatedly fails to respond to disclosure, children withdraw trust from institutions and reinvest it in particular individuals, so that institutional trust is weakened, not simply absent. Subtheme E points to a stronger version of this dynamic. Where the formal pathway not only fails to help but rebounds on the child — through parents’ standing as legal guardians, an evidentiary threshold that is rarely met, or a school exposed to family backlash — the experience approaches institutional betrayal (35), the distinct harm that arises when an institution on which a person depends fails to protect them or compounds the harm they have suffered. The distinction is analytically useful: most of our themes describe institutional trust left unreinforced, a passive gap that a responsive service could fill, whereas Subtheme E describes trust actively injured, which is harder to repair. Label avoidance (23, 24) operates as a parallel mechanism at the service tier. The contribution is to show how these mechanisms interlock in a fragmented, low-resource child-protection system, so that a service-gap map and a trust analysis, read together, explain why expanding detection without expanding response is counterproductive.

This has a direct implication for the ethics of screening as public health practice. This is not a new principle so much as a neglected one: the classic criteria for a screening programme (36) require that an accepted treatment and the facilities to deliver it be in place before screening is introduced, so that case-finding is never divorced from care. School-based detection is, in this sense, a screening programme, and one that identifies need without an assured, acceptable, and accessible service at its output does not meet that long-standing test. Expanding detection without a reliable, accessible, and non-stigmatizing service at the output does not just fail to help; it can cause secondary harm because it repeatedly signals to children that disclosure changes little and can expose the child and the adults who try to help to further difficulty. In equity terms, the burden of the gap falls hardest on children least able to navigate a fragmented system independently, which places the problem within the concerns of Sustainable Development Goals 3 and 10.

*This unevenness is patterned*. Rural students in our study were furthest from the single provincial mental-health facility and least likely to have independent access to a phone or to information about the helpline (Subthemes C2 and D). Children harmed by a parent faced a formal pathway blocked at the guardianship step (Subtheme E). Those already anticipating a stigmatizing label were the most likely to avoid the one specialist service that existed (Subtheme C2). Where detection is scaled up, but response is not, these differences compound: screening identifies more children precisely among the groups for whom no usable route onward exists, so an intervention meant to protect can widen the very inequities it aims to close. There is a further, subtler inequity in the substitution of personalized for institutional trust. Trust vested in a particular teacher does not scale and cannot be guaranteed: it depends on a child happening to encounter an adult who is both willing and able to help — unevenly distributed across schools, and what over-stretched, under-professionalized settings cannot assure. A system that leans on personalized trust to compensate for absent institutional trust therefore protects children according to the relationships they happen to have rather than the entitlements they hold.

*This dilemma is most severe when the abuser is a parent or caregiver*. Reporting the abuse forces the child, or a teacher helping them, to take action against the family the child relies on. This risks potential retaliation, while actual protection remains uncertain. Furthermore, the official system is limited. While extreme cases may face criminal prosecution, much of the everyday abuse — which participants viewed as normalized — is handled through administrative fines. Under Decree 130/2021, fines range from 10 to 20 million VND (approximately USD 400–800) for various forms of child abuse (37). This penalty usually leaves the child in the same household and rarely improves the situation.

Consequently, the risks often outweigh the benefits: reporting causes real damage to family relationships, while the legal outcome is often minimal. This difficulty, alongside a lack of available support services, explains why official reporting channels are frequently avoided even when they exist.

Although grounded in one province and not generalizable in a statistical sense, the mechanism we describe may not be confined to it. Many low- and middle-income systems are expanding school-based detection of mental-health and protection needs faster than they are building the referral, treatment and follow-up capacity that detection presupposes (14), and some share the structural features that made the gap consequential here: responsibility dispersed across ministries, a single visible helpline standing in for a full service tier, and specialist mental-health care concentrated in distant, stigmatized facilities. Where those conditions hold, a similar dynamic — detection outpacing response, and unanswered disclosure eroding institutional trust — could plausibly arise. We offer this as a hypothesis for other settings to test rather than a claim about them. The value of screening is bounded by the services it can hand children on to, so system investment should lead the expansion of detection rather than follow it.

### 5.1 The 2025 reform: risks and opportunities

We do not evaluate the reform empirically — our fieldwork ended in March 2024, before it took effect — but discuss its potential implications, which post-reform studies should test. The 2025 reform is double-edged. In the short term, it weakened the early stage of the chain: abolishing the district tier removed an intermediate coordinating layer, and because the corresponding services are not yet in place, the transition can leave children with even fewer clear destinations. As responsibility is pushed down to the commune level, commune social-welfare officials — who directly receive child-violence cases — are likely to carry a heavier load with uneven professional capacity, making the commune the new bottleneck in the safety net. In the longer term, however, moving child-related functions into the health sector could open an opportunity the previous arrangement lacked, namely to embed social-work and psychosocial services within the health system along its established tiers, giving post-screening support a clearer institutional home. Whether the reform ultimately narrows or widens the gap therefore depends on implementation: the opportunity is realized only if social-work and coordination functions are actively built into the new structure, and the risk is that they are displaced by a purely medical reading of what are, in part, social and relational problems.

*This has a direct bearing on how the reform should be evaluated*. The pre-reform system’s central weakness was a deficit of institutional trust, not of administrative structure alone. Although our qualitative data were collected before the reform (December 2023 to March 2024), they diagnose a trust gap that the 2025–2026 transition inherits; our reading of the reform is therefore prospective and rests on the desk review. The implication is that the reform should be judged by whether it repairs that trust — by rebuilding a visible, responsive post-screening pathway that children and teachers can rely on — rather than by whether it merely streamlines the management model. Reorganizing who administers the system, without restoring the response that disclosure meets, would leave the trust gap intact, and could deepen it during a prolonged transition.

A more immediate policy signal is the Ministry of Education and Training’s 2025 circular on school counselling and social work (38), which formalizes school-counselling and social-work functions in general education schools. Two cautions follow from our findings. First, as with the administrative reform, issuing a policy is not the same as staffing it: in our schools the role was still carried by teachers or administrative staff with only a few days’ training, so the circular’s benefit will depend on whether qualified personnel, supervision and referral links actually follow. Second, because full implementation will take time, interim measures are needed now — giving existing teacher-counsellors structured training and supervision, setting clear referral protocols to commune and health services, and using the national helpline as a temporary bridge — so that children identified today are not left without a usable response while the professional workforce is built.

### 5.2 Policy implications

The practical direction follows the chain itself. First, make one actor accountable for the post-screening stage, and pair a clear referral pathway with a feedback loop back to the student. Second, embed accessible, non-stigmatizing psychosocial and mental-health support close to schools — care that is approachable, acceptable and available at the point of need (39, 40) — rather than at a distant specialist hospital, by designating a small set of child- and adolescent-friendly referral points to which schools can send students identified after screening, and re-establish a local coordination point after the district tier’s abolition. Third, build a routine mechanism for students to be heard. Fourth, widen and actively publicize the ways children can reach the one near-universal channel that already exists. Awareness campaigns have centered on telephoning 111, so children — who often have no private phone — may not call. The helpline can also be reached through a dedicated mobile app and messaging platforms such as Zalo, which are easier to access and more private, and so better suited to disclosure, but of which students and teachers alike are often unaware (32). Communication in schools should therefore publicize the full range of ways to reach 111, not the telephone alone, targeting both students and teachers, so that the phone barrier our participants described is not, on its own, decisive. The message for policy is not to slow detection but to match it with capacity: our findings suggest that screening should be expanded only alongside the capacity to refer children onward.

### 5.3 Limitations

These findings should be read against their limits. The desk review depends on the quality and completeness of available records, and the reform is still in a 2025–2026 transition, so the map describes a moving target. Crucially, the interviews were conducted between December 2023 and March 2024, before the reform, so they describe the pre-reform system; our account of the reform rests on the desk review and is prospective, and its actual effects on children — adverse in the short term or beneficial in the longer term — will require post-reform empirical study. The qualitative interviews were primary but purposive and bounded to specific settings; they are intended to illuminate mechanisms, not to estimate how common those mechanisms are. The aim throughout is transferability — the account should be judged by how well it travels to other child-protection systems with similar fragmentation — rather than statistical generalization (29).

As the fieldwork was concentrated in a single province, the findings are not statistically generalizable, and their value lies in analytical rather than statistical transferability. The mechanisms we describe — screening running ahead of services, and trust vested in individuals rather than institutions — are tied to structural features of the Vietnamese public system that are shared well beyond the study site: a thin child mental-health workforce, typically one specialized psychiatric facility per province, no non-stigmatizing clinical-psychology alternative at general hospitals, and school counselling delivered as an added teacher duty. We therefore provide detailed contextual information (Table 2; Section 3.2) so that readers can judge how far these mechanisms transfer to other Vietnamese provinces and to comparable lower-resource settings, rather than claiming that the sample represents the country.

*A further boundary concerns school type*. Our sites were public lower-secondary schools, and the findings reflect that public system, in which counselling is typically an additional duty assigned to a teacher rather than a dedicated professional role. Private schools may employ their own psychological staff and could differ in this respect; the study province, however, had no private schools, so this possibility lies outside our data.

### 5.4 Conclusion

Screening in this setting is itself thin — relatively few children are formally detected — and, crucially, even where detection does occur, the path to support is not straightforward. Detection therefore runs ahead of services in two senses: it is limited in reach, and what little it achieves rarely converts into protection. The fragmented post-screening gap, which the 2025 reform may have compounded, appears to act as a structural driver that leaves students’ trust unreinforced and, in the strongest cases, erodes it. The message for policy and public health is that our findings caution against expanding detection faster than the capacity to refer children onward, and against assuming that detection alone secures protection. Although based in one province, the mechanism is likely to travel to other low- and middle-income settings that are scaling up school-based screening without matching investment in referral and follow-up. Future research should test this prospectively, follow children through the post-2025 system, and evaluate whether embedding social-work and coordination functions in the new structure restores the response that detection assumes.

## Data Availability

The datasets presented in this article are not readily available because the qualitative datasets are not publicly available to protect the privacy and confidentiality of minor participants; de-identified excerpts are available from the corresponding author on reasonable request, subject to ethics-committee approval. Requests to access the datasets should be directed to lmt@huph.edu.vn.

## Supplementary material

Supplementary Table S1 Core authoritative sources reviewed in the national desk review, by governance line and relevance.

Supplementary Table S2 Coding framework: overarching themes, subthemes and representative codes

COREQ checklist Consolidated Criteria for Reporting Qualitative Research (COREQ): 32-item checklist for interviews and focus groups.
