# Supplementary material for: Screening with a limited safety net: post-screening service gaps and inequitable access to child protection in Vietnam
Source: Front Public Health. 2026 Oct 21;14:1930066. doi: 10.3389/fpubh.2026.1930066 (PMC13731252; doi:10.3389/fpubh.2026.1930066)
Supplement: Supplementary file 1 [file Supplementary_file_1.docx]

# **Supplementary Material — COREQ checklist**

Consolidated Criteria for Reporting Qualitative Research (COREQ): 32-item checklist for the manuscript *“Screening with a limited safety net: Post-screening service gaps and inequitable access to child protection in Vietnam.”*

Checklist developed from Tong A, Sainsbury P, Craig J. Consolidated criteria for reporting qualitative research (COREQ): a 32-item checklist for interviews and focus groups. Int J Qual Health Care (2007) 19:349–357 (reference 31). “Reported” = Yes / Partial / No; section numbers refer to the main manuscript.

| **No.** | **COREQ item** | **Reported** | **Where addressed in the manuscript** |
| --- | --- | --- | --- |
| **Domain 1: Research team and reflexivity** | | | |
| 1 | Interviewer/facilitator | Yes | Two trained female researchers conducted the sessions (Section 3.2). |
| 2 | Credentials | Yes | One interviewer holds a PhD and the other a Master’s degree; public-health/social-work researchers (Sections 3.2, 3.4). |
| 3 | Occupation | Yes | Academic researchers external to the participating schools (Section 3.4). |
| 4 | Gender | Yes | The two interviewers were female (Section 3.2). |
| 5 | Experience and training | Yes | Both interviewers were experienced in qualitative research (Section 3.2); reflexivity noted (Section 3.4). |
| 6 | Relationship established | Yes | Researchers were external to the schools, with no prior relationship to participants (Section 3.4). |
| 7 | Participant knowledge of the interviewer | Partial | Participants were informed of the study's purpose during consent (Section 3.5). |
| 8 | Interviewer characteristics | Yes | Reflexivity statement on the researchers' positions and possible influence (Section 3.4). |
| **Domain 2: Study design** | | | |
| 9 | Methodological orientation and theory | Yes | Convergent health-systems study with a qualitative-descriptive core; reflexive thematic analysis, abductive (Sections 3.1, 3.3). |
| 10 | Sampling | Yes | Purposive sampling across eight lower-secondary schools (four rural, four urban) (Section 3.2). |
| 11 | Method of approach | Yes | Recruited through written invitation letters; only invited individuals took part (Section 3.2). |
| 12 | Sample size | Yes | 25 in-depth interviews (including one provincial education-authority leader) and 16 focus group discussions (Section 3.2; Table 2). |
| 13 | Non-participation | Partial | Recruitment was by written invitation; refusals/drop-outs were not separately quantified. |
| 14 | Setting of data collection | Yes | Schools in Bắc Giang province, Vietnam (Section 3.2). |
| 15 | Presence of non-participants | Yes | No third parties present; only the invited participants and the researchers (Section 3.2). |
| 16 | Description of sample | Yes | Participant characteristics in Table 2 (Section 3.2). |
| 17 | Interview guide | Yes | A semi-structured guide informed by the two conceptual lenses was used (Sections 3.2, 3.3); pilot-testing not reported. |
| 18 | Repeat interviews | Yes | No repeat interviews; a summary was fed back at the end of each session (Sections 3.2, 3.4). |
| 19 | Audio/visual recording | Partial | Sessions were transcribed, implying recording; the recording method is not explicitly stated (Section 3.4). |
| 20 | Field notes | Yes | Field notes were taken during the sessions (Section 3.2). |
| 21 | Duration | Yes | Interviews and focus groups lasted approximately 45–60 minutes (Section 3.2). |
| 22 | Data saturation | Yes | Adequacy judged by information power rather than saturation (Section 3.3). |
| 23 | Transcripts returned | Yes | Transcripts not returned; end-of-session summaries invited comment/correction (respondent validation) (Section 3.4). |
| **Domain 3: Analysis and findings** | | | |
| 24 | Number of data coders | Yes | Collaborative team coding with reflexive dialogue between coders (Sections 3.3, 3.4). |
| 25 | Description of the coding tree | Yes | Thematic structure built and reported as themes/subthemes in Table 3 (Sections 3.3, 4.2). |
| 26 | Derivation of themes | Yes | Abductive: deductive codes from the two lenses plus inductive codes from the data (Section 3.3). |
| 27 | Software | Yes | Mindjet MindManager used to organise codes and build the thematic structure (Section 3.3). |
| 28 | Participant checking | Partial | End-of-session summaries were checked with participants (Section 3.4); feedback on the final findings was not sought. |
| 29 | Quotations presented | Yes | Illustrative quotations identified by source (IDI/FGD, role) in the Results and Table 3 (Section 4.2). |
| 30 | Data and findings consistent | Yes | Findings are grounded in and consistent with the quoted data (Section 4.2). |
| 31 | Clarity of major themes | Yes | Two overarching themes clearly presented (Table 3; Section 4.2). |
| 32 | Clarity of minor themes | Yes | Subthemes and diverse cases discussed (Section 4.2; Table 3). |
