# Supplementary material for: Screening with a limited safety net: post-screening service gaps and inequitable access to child protection in Vietnam
Source: Front Public Health. 2026 Oct 21;14:1930066. doi: 10.3389/fpubh.2026.1930066 (PMC13731252; doi:10.3389/fpubh.2026.1930066)
Supplement: Supplementary file 2 [file Table_1.docx]

# **Supplementary Material**

For the manuscript *“Screening with a limited safety net: Post-screening service gaps and inequitable access to child protection in Vietnam.”*

## **Supplementary Table S1 — Core authoritative sources reviewed in the national desk review, by governance line and relevance**

| **Source** | **Type** | **Relevance to the review** | **Ref.** |
| --- | --- | --- | --- |
| Law on Children (2016) | Legislation | Distributes child-protection responsibilities across agencies | (15) |
| Decree 56/2017 (detailing the Law on Children) | Legislation | Levels of child protection (prevention, support, intervention); inter-agency responsibilities | (16) |
| Resolution 202/2025 (provincial administrative units) | Legislation | 2025 administrative reform; coordination architecture | (18) |
| Circular 18/2025 (school counselling and social work) | Policy | School screening and counselling function | (38) |
| Multiple Indicator Cluster Survey, Vietnam 2014 | National survey | Prevalence of violent discipline | (1) |
| Global School-based Student Health Survey, Vietnam 2013 | National survey | Adolescent mental-health need | (6) |
| Viet Nam National Adolescent Mental Health Survey (V-NAMHS) | National survey | Adolescent service use and unmet need | (7) |
| UNICEF Vietnam and ODI (2018) | Agency report | MH/psychosocial services; stigma; fragmentation | (17) |
| National Child Helpline 111 — annual call statistics | Operational data | Helpline reach and onward referral | (32) |
| UNICEF EAP, UNFPA and UN Women (2020) | Agency report | Referral/follow-up gaps; evidence and justice barriers | (33) |

*Repositories searched: National Assembly legal database; portals of the Ministries of Health, of Labour – Invalids and Social Affairs, and of Education and Training; national survey report archives; and international-agency (UNICEF, WHO, UNFPA, UN Women, ODI) publications on Vietnam. All references correspond to the numbered reference list of the main manuscript.*
