# Supplementary material for: Screening with a limited safety net: post-screening service gaps and inequitable access to child protection in Vietnam
Source: Front Public Health. 2026 Oct 21;14:1930066. doi: 10.3389/fpubh.2026.1930066 (PMC13731252; doi:10.3389/fpubh.2026.1930066)
Supplement: Supplementary file 3 [file Table_2.docx]

# **Supplementary Material**

For the manuscript *“Screening with a limited safety net: Post-screening service gaps and inequitable access to child protection in Vietnam.”*

## **Supplementary Table S2 — Coding framework: overarching themes, subthemes and representative codes**

| **Subtheme** | **Representative codes** | **Description** |
| --- | --- | --- |
| **Overarching theme 1 — Help-seeking pathway: disclosure stalls at its pivotal link** | | |
| A — “Nowhere to go” | no counselling or medical room at school; teachers untrained in psychosocial support; counsellor role hollowed out by staff streamlining (role taken by a Youth Union teacher); no onward referral destination after disclosure | A concern is recognised at school but no accessible service follows at the point of access. |
| C1 — Family-level barriers (parent is the source of harm) | harm treated as a private family matter; disclosure only when harm becomes extreme; confiding limited to close friends; normalisation of physical punishment (“spare the rod”); loyalty and fear of consequences | Barriers to disclosing intrafamilial harm sit at the family level rather than at the service tier. |
| E — Disclosure rebounds; the formal response stalls | parents invoke guardianship / “family’s business”; school exposed to family backlash; cases stall for want of visible evidence; disclosure exposes the child and helping adults to further difficulty | Pursuing a case through the formal pathway can expose rather than protect (institutional betrayal). |
| **Overarching theme 2 — Institutional trust (with stigma and label avoidance)** | | |
| B — “Speaking up changes little” (unanswered disclosure) | disclosure meets no visible response or feedback; “no one who can truly help”; institutions do not reliably act; trust left unreinforced rather than actively destroyed | Unanswered disclosure signals that the institution will not reliably respond. |
| D — Trusting people, not the system | limited rural access to the 111 helpline; few other support services; trust invested in particular individuals; routing to informal sources (friends, the internet) | Children rely on trusted individuals — typically peers and friends — rather than on the formal system or formal channels. |
| C2 — Label avoidance (the “mental” label) | stigma of the local clinical term for mental illness (connoting “crazy”); fear of ridicule (“friends would laugh”); avoiding a referral to avoid the label; single provincial psychiatric hospital understood to serve severe illness; no non-stigmatising clinical-psychology alternative at district/provincial general hospitals, so the psychiatric hospital is the only formal destination | A mental-health referral is avoided because of the label attached to the service; with no low-stigma alternative within reach, seeking help and accepting the label become the same choice. |

*Codes were developed abductively (deductive codes from the two conceptual lenses plus inductive codes from the data) and organised into subthemes A–E and two overarching themes. Subtheme C is split into family-level barriers (C1) and label avoidance at the service tier (C2). Subtheme labels correspond to Table 3 of the main manuscript.*
